# Rhabdomyolysis and Acute Kidney Injury: Exploring the Potential Causes in a Hospitalized Patient

**DOI:** 10.7759/cureus.80535

**Published:** 2025-03-13

**Authors:** Pratap Kumar Upadrista, Sindhu Harika Peketi, Nagakowsikreddy Sudireddy, Bair Cadet, Zae Kim

**Affiliations:** 1 Nephrology, Northwell Health, East Meadow, USA; 2 Internal Medicine, Nassau University Medical Center, East Meadow, USA; 3 Nephrology, Nassau University Medical Center, East Meadow, USA

**Keywords:** neuroleptic malignant syndrome (nms), non-traumatic rhabdomyolysis, rhabdomyolysis aki, serotonin syndrome diagnosis, venlafaxine rhabdomyolysis

## Abstract

Rhabdomyolysis refers to the breakdown of skeletal muscle with subsequent release of intracellular contents into blood, causing elevated creatine kinase (CK) and renal damage. The four main etiological categories are traumatic, non-traumatic, non-traumatic exertional, and non-traumatic non-exertional. We report a case of a young female who presented with rhabdomyolysis due to agitation, increased motor activity, and venlafaxine use, resulting in severe organ dysfunction. A 30-year-old female on venlafaxine, pregabalin, and fentanyl patch presented to our hospital with a sudden onset of generalized pain and agitation. Before presentation, the patient was flailing her extremities and thrashing herself against the wall, and the patient became more confused, which prompted the mother to bring her to our emergency department. She had icterus, diffuse ecchymosis over bilateral upper and lower extremities, edematous extremities, and generalized weakness of all extremities with 3/5 strength, normal reflexes without rigidity or clonus or tremors. At presentation, the patient had elevated CK, elevated creatinine, transaminitis, international normalized ratio (INR) of 5.4, and ammonia of 126 umol/L. Given rhabdomyolysis and non-oliguric acute kidney injury, the patient was started on aggressive intravenous hydration at admission. However, her creatinine worsened in the next 24 hours, and the patient developed a worsening of creatinine with high anion gap metabolic acidosis. The patient was given a Lasix challenge and sodium bicarbonate infusion to aid in the excretion of myoglobin and N-acetylcysteine infusion for worsening liver function. She became anuric despite diuretics, developed acute tubular necrosis, and was started on intermittent hemodialysis. Over the next few days, her urine output improved and her creatinine gradually improved. During her hospital course, her mental status returned to baseline, she was started on physical therapy as tolerated, her lower extremity strength gradually improved, and she was discharged to an inpatient rehabilitation facility. Agitation is one of the most common presentations to the emergency department and can cause rhabdomyolysis. Careful monitoring, evaluation of other coexisting causes of rhabdomyolysis, and early aggressive hydration are of paramount importance. Additionally, the use of prediction tools such as the McMahon score can aid in identifying patients who will need renal replacement therapy.

## Introduction

Rhabdomyolysis refers to the breakdown of skeletal muscle with subsequent release of intracellular contents into blood, causing elevated creatine kinase (CK) and renal damage. The four main etiological categories are traumatic, non-traumatic, non-traumatic exertional, and non-traumatic non-exertional [1]. We report a case of a young female who presented with rhabdomyolysis due to agitation, increased motor activity, and venlafaxine use resulting in severe organ dysfunction.

## Case presentation

A 30-year-old female, with a past medical history significant for endometriosis and adenomyosis requiring multiple exploratory laparotomy surgeries, anxiety, and depression on venlafaxine, nerve compression at multiple levels requiring pregabalin and fentanyl patch, presented to our hospital with sudden onset of generalized pain and agitation. As per the mother, the patient managed her prior episodes of tremendous pain by flailing her extremities and thrashing herself against the wall. However, this episode lasted longer, and the patient became more confused, which prompted the mother to bring her to our emergency department. The patient takes medical marijuana for managing her pain and is not on any other recreational drugs. On examination, she was confused and was groaning in pain. She was afebrile and had sinus tachycardia of 110 beats per minute, blood pressure of 116/77 mmHg, respiratory rate of 20/minute, and saturating at 95% on room air. She had scleral icterus, diffuse ecchymosis over bilateral upper and lower extremities, and edematous extremities but had good peripheral pulses with normal capillary refill. She had generalized weakness in all extremities with 3/5 strength and normal reflexes. She did not have rigidity or clonus or tremors.

At presentation, the patient had elevated CK, elevated creatinine, and blood urea nitrogen, transaminitis, elevated international normalized ratio (INR), and ammonia (as depicted in Table 1).

**Table 1:** The patient's labs on admission

Parameter	Value on Admission	Normal Reference Range
Creatine Kinase	15,626 U/L	34-145 U/L
Creatinine	2 mg/dL	0.6-1.0 mg/dL
Blood Urea Nitrogen (BUN)	57 mg/dL	9-23 mg/dL
Alanine Transaminase (ALT)	473 U/L	7-40 U/L
Aspartate Transaminase (AST)	2139 U/L	13-40 U/L
Alkaline phosphatase (ALKP)	127 U/L	43-86 U/L
Total Bilirubin	0.9 mg/dL	0.3-1.2 mg/dL
INR	5.4	0.8-1.1
Ammonia	126 umol/L	11-32 umol/L

The serum tox screen is negative for acetaminophen, ethanol, and salicylate, while her urine detox panel was positive for cannabinoids and benzodiazepines. However, the patient received lorazepam in the emergency department due to agitation. Her urinalysis was suggestive of hematuria, which may be suggestive of traumatic Foley insertion. Given rhabdomyolysis and non-oliguric acute kidney injury (AKI), the patient was started on aggressive intravenous hydration at admission. However, her creatinine kinase worsened in the next 24 hours and reached a peak of 186,664 U/L. The patient developed a worsening creatinine to 7.8 mg/dL with high anion gap metabolic acidosis and transaminitis (alanine aminotransferase (ALT) of 3900 U/L and aspartate transaminase of 4200 U/L) with jaundice (total bilirubin of 10.3 mg/dL and direct bilirubin of 7.9 mg/dL).

**Figure 1:**
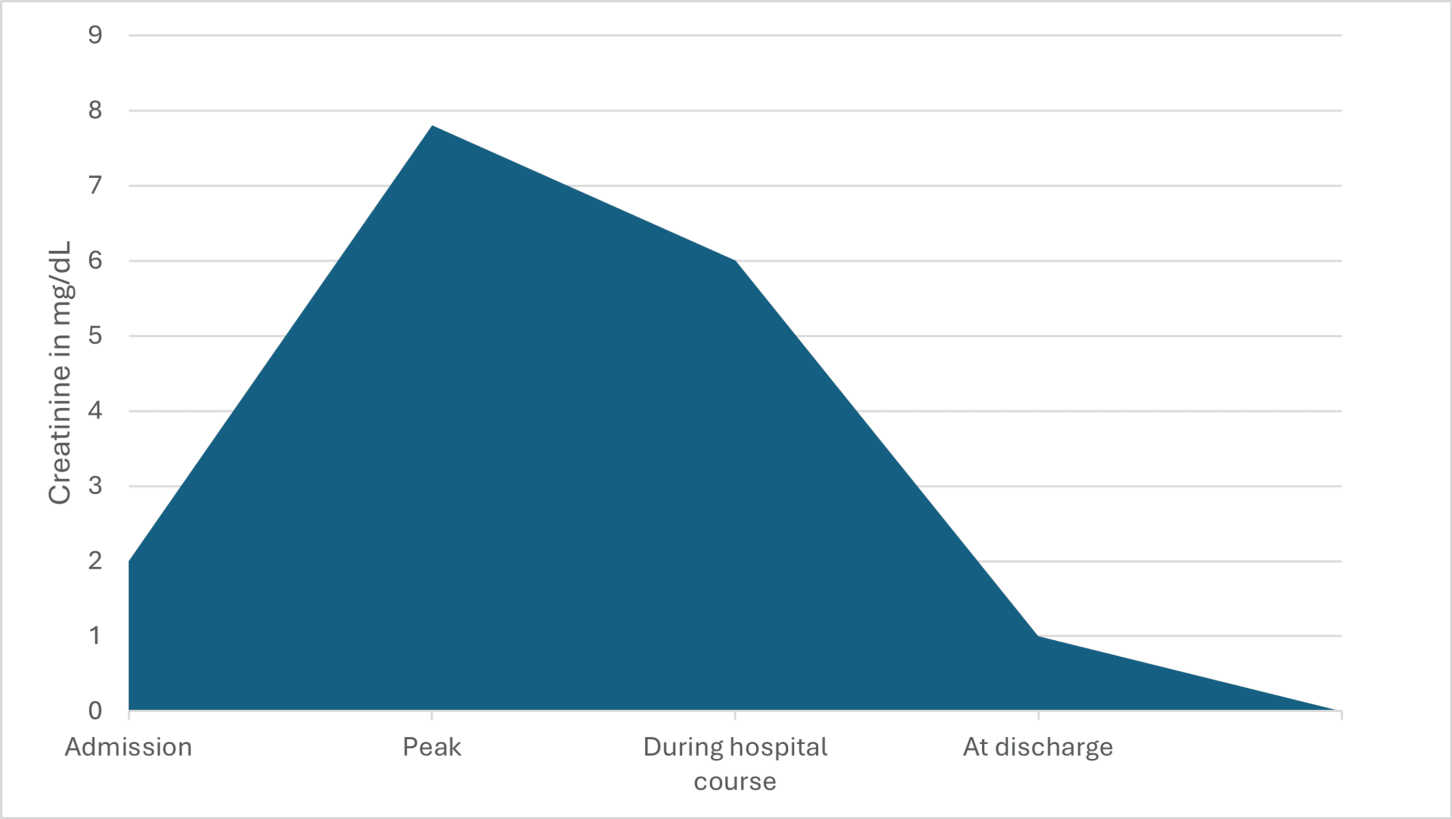
Trend of AKI during hospital stay in our patient with rhabdomyolysis AKI: acute kidney injury

Given worsening liver and renal function in the setting of extensive rhabdomyolysis and encephalopathy, the patient was transferred to the intensive care unit for closer monitoring. The patient was given a Lasix challenge and sodium bicarbonate infusion to aid in the excretion of myoglobin and N-acetylcysteine infusion for worsening liver function and was considered for a liver transplant. Over the next few hours, her transaminitis, hyperbilirubinemia, and coagulopathy decreased, and encephalopathy was presumed to be multifactorial, which improved gradually. The electroencephalogram showed diffuse slow waveforms. However, creatinine continued to worsen, and the patient’s urine output decreased. She became anuric despite diuretics as she developed acute tubular necrosis (ATN) and was started on intermittent hemodialysis. Over the next 10 days, the patient received intermittent hemodialysis, her urine output improved, and her creatinine gradually improved. After the initial decline of creatinine, there was a plateau of 6-7 mg/dL for the next week, with a subsequent decline in the creatinine to 1.0 mg/dL. Hemodialysis was discontinued (trend shown in Figure 1).

During her hospital course, her mental status returned to baseline, and she was started on physical therapy as tolerated. Her lower extremity strength gradually improved, and she was discharged to an inpatient rehabilitation facility.

## Discussion

Rhabdomyolysis can be potentially classified into three categories based on the mechanism of injury [1]. It can be traumatic due to muscle compression, nontraumatic exertional, and nontraumatic non-exertional due to various drugs (e.g., selective serotonin reuptake inhibitors (SSRI), serotonin-norepinephrine reuptake inhibitors (SNRI), monoamine oxidase inhibitors (MAOIs), tricyclic antidepressants, synthetic opioids, antipsychotic, or sudden cessation of dopaminergic agents causing serotonin syndrome and neuroleptic malignant syndrome) or toxins, infections, or electrolyte disturbances [1]. Any variety of these events can trigger cell death, which will increase intracellular free ionized cytoplasmic and mitochondrial calcium. This increased intracellular calcium leads to protease activation, increased skeletal muscle cell contractility, mitochondrial dysfunction, and production of reactive oxygen species, all of which result in skeletal muscle cell death [2]. Myocyte death triggers the release of intracellular contents such as CK and other muscle enzymes, myoglobin, and electrolytes.

Myoglobin is filtered by the glomerulus into the urinary space where it is degraded and releases heme pigment. The release and subsequent deposition of myoglobin in the kidneys result in acute renal failure (ARF) in up to 40% of cases of rhabdomyolysis and account for 7% of cases of ARF in the US [3]. The primary mechanisms for renal failure due to myoglobin are renal vasoconstriction and tubular obstruction, but the most important mechanism might be lipid peroxidation and tubular injury [3]. However, two crucial factors are significant in the development of ARF due to myoglobinuria: hypovolemia/dehydration and acidic urine. In the absence of these factors, myoglobin has minimal nephrotoxic properties [4]. While there is no absolute cut-off value for CK elevation to diagnose rhabdomyolysis, marked elevation of CK (typically five times the upper limit of normal) in the context of triggering factors and characteristic features such as myalgia, weakness, and dark urine aid in the diagnosis of rhabdomyolysis. They may have associated hypovolemia from third-spacing due to the influx of extracellular fluids into injured muscles. Patients can also develop cardiac dysrhythmia and reversible liver dysfunction. Laboratory findings are significant for hyperkalemia, hyperphosphatemia, and severe hyperuricemia due to the release of intracellular contents and hypocalcemia in the initial stages due to calcium deposition in damaged muscles, AKI, and high anion gap metabolic acidosis [5]. Fractional excretion of sodium is lower in ATN due to rhabdomyolysis as compared to other forms of ATN due to renal vasoconstriction and concurrent volume depletion [6]. In addition to CK elevation, other markers of muscle injury such as aldolase, aminotransferases, and lactate dehydrogenase are also elevated.

Dark-colored urine and a positive dipstick test in the absence of any visible red cell by microscopy are suggestive of myoglobinuria. Varying degrees of proteinuria may also be found on urinalysis. Heme pigment combines with Tamm-Horsfall protein and produces characteristic pigmented granular casts on urine microscopy [4].

In 2013, McMahon et al. [7] developed a prediction tool for the poor outcomes and need for renal replacement therapy in patients with rhabdomyolysis. It is based on gender, age of the patient, initial creatinine, calcium, CK, phosphate, bicarbonate, and the reason for rhabdomyolysis. Our patient had a McMahon score of 6, which suggests that the patient might need renal replacement therapy with 86% sensitivity.

The mainstay of AKI prevention in patients with rhabdomyolysis is early and aggressive volume hydration with intravenous crystalloids such as isotonic saline and titration of the fluids is based on the patient’s volume status and urine output [8]. Patients with rhabdomyolysis might benefit from alkalinization of urine with bicarbonate infusion, especially in patients with CK above 5,000 U/L or in those with clinical evidence of severe muscle injury and rapidly rising CK levels. Bicarbonate infusion is titrated to achieve a urine pH of 6.5 with monitoring of arterial pH and serum calcium. A forced alkaline urine diuresis, in theory, prevents pigment precipitation and release of free iron and diminishes kidney toxicity [9-11]. Once acute renal failure has developed, the patient requires hemodialysis. There is no role of dialysis in preventing AKI.

Our patient had extreme agitation and flailing episodes to control her pain, which could have contributed to non-traumatic exertional rhabdomyolysis. Given that our patient was on SNRI (venlafaxine) and fentanyl at home with no recent change in dose and received haloperidol in the emergency department to control her agitation, we considered serotonin syndrome and neuroleptic malignant syndrome. The patient had encephalopathy on presentation but did not have rigidity, hyperreflexia, clonus, autonomic dysfunction, or fevers to support either of the diagnoses. While neuroleptic malignant syndrome and serotonin syndrome can contribute to rhabdomyolysis and subsequent AKI, our patient did not have other features to support these diagnoses.

## Conclusions

Agitation is one of the most common presentations to the emergency department and can cause rhabdomyolysis. Careful monitoring, evaluation of other coexisting causes of rhabdomyolysis, and early aggressive hydration are of paramount importance. Additionally, the use of prediction tools such as the McMahon score can aid in identifying patients who will need renal replacement therapy.
